# Relationship between health literacy and COVID-19 knowledge: A cross-sectional study

**DOI:** 10.3389/fpubh.2023.1058029

**Published:** 2023-02-20

**Authors:** Zhenbo Tao, Qianqian Xu, Yingying Zhu, Qiuhong Mei, Hongwei Feng, Qiuyan Jin, Shige Ding, Ying Dong

**Affiliations:** Ningbo Municipal Center for Disease Control and Prevention, Ningbo, Zhejiang, China

**Keywords:** health literacy, COVID-19, knowledge, attitude, behavior

## Abstract

**Background:**

Health literacy (HL) is a protective factor for some chronic diseases. However, its role in the Coronavirus Disease 2019 (COVID-19) pandemic has not been clarified. This study aims to explore the association between HL and COVID-19 knowledge among residents in Ningbo.

**Methods:**

A total of 6,336 residents aged 15–69 years in Ningbo were selected by multi-stage stratified random sampling method. The “Health Literacy Questionnaire of Chinese Citizens (2020)” was used to evaluate the relationship between COVID-19 knowledge and HL. Chi-square test, Mann-Whitney *U* test and logistic regression were used to analyze the data.

**Results:**

The HL and COVID-19 knowledge levels of Ningbo residents were 24.8% and 15.7%, respectively. After adjusting for confounding factors, people with adequate HL were the more likely to have adequate COVID-19 knowledge compared with those with limited HL (*OR* = 3.473, 95% CI = 2.974–4.057, *P* <0.001). Compared with the limited HL group, the adequate HL group had a higher rate of COVID-19 knowledge, a more positive attitude, and a more active behavior.

**Conclusion:**

COVID-19 knowledge is significantly associated with HL. Improving HL may influence people's knowledge about COVID-19, thereby changing people's behaviors, and finally combating the pandemic.

## Introduction

On December 31, 2019, the International Committee on Classification of Viruses isolated a novel coronavirus strain from patients with pneumonia of unknown cause in Wuhan and named it SARS-CoV-2 (1). On 11 March 2020, the World Health Organization (WHO) declared COVID-19 a “Public Health Emergency of International Concern” (2). In terms of geographical scope, number of infected persons, and world economic recession, the impact of COVID-19 is shocking. As of June 3, 2022, COVID-19 has infected at least 528 million people and killed 6 million (3). The current COVID-19 pandemic poses a huge threat to global public health, people's lives and the world economy.

In the face of an outbreak of a major infectious disease, no individual can be immune. It is crucial for residents to master relevant knowledge, have a positive attitude and take appropriate actions to prevent and control the epidemic. The rapid development of COVID-19 requires people to access and use health information to adjust their behavior at a fast pace (4). COVID-19 prevention measures, such as frequent hand washing and wearing masks, have been clarified. But the effective implementation of these measures has been affected by perceptions (5). Public knowledge is essential to prevent the spread of COVID-19 due to the lack of effective treatment measures, and large-scale public knowledge campaigns have played a key role in the fight against COVID-19 (6).

A study has shown that race/ethnicity, education, and socioeconomic status are associated with COVID-19 knowledge (7). However, there are few studies on health literacy (HL) and COVID-19 knowledge. Health literacy is an individual's ability to obtain, understand, evaluate and use information to make decisions and take actions that have an impact on health status (8). Health literacy questionnaires are different in different countries. The Canadian HL questionnaire includes 191 questions about daily life, covering five dimensions: health promotion, health protection, disease prevention, systems orientation, health and disease management (9). HL in the United States was measured with a modified version of the Single-Item Health Literacy Screener, designed to measure limited reading ability, a principal component of HL (10). In China, the HL questionnaire includes three dimensions: knowledge and attitudes, behavior and lifestyle, and health-related skills. One study found that improving HL was effective in helping residents prevent and control disease (11).

As far as we know, the relationship between residents' HL and knowledge, attitude and behavior of COVID-19 prevention and control has not been reported. Understanding this relationship can not only improve the awareness of the importance of HL of the whole society, but also facilitate the prevention and control of major infectious diseases. Hence, based on the 2020 China Health Literacy Survey, this study collected relevant data from Ningbo residents, and explored the relationship between residents' HL and knowledge, attitude and behavior of COVID-19 prevention and control.

## Methods

### Study population

This study was a cross-sectional study of people aged 15–69 years who had lived continuously in Ningbo for more than 6 months. This study was reviewed and approved by the Research Ethics Committee of the Ningbo Municipal Center for Disease Control and Prevention (Approval No.: 202203). All potential participants had read and understood the consent information and agreed to the questionnaire.

### Sampling methods

The minimum sample size for each county (district) was calculated using the formula *N* = $\frac{{\mu_{\alpha }}^{2*}p^{*}\left(1-p\right)}{\delta^{2}}$^*^deff. Based on the HL of Zhejiang Province in 2019, the level of HL was 29.49%, *p* = 0.2949, the allowable relative error was set to 15%, and the allowable absolute error δ = 0.2949 ^*^ 0.15 = 0.0442, μ_α_ = 1.96, *deff* = 1. The minimum sample size for each layer was 408. The sample size was set at 640 per county (district) to account for invalid questionnaires and rejection rates. Stratified multistage probabilities proportional to population size sampling was used in this study. The whole sampling was divided into four stages: (1) Four townships were selected from each of the 10 counties (districts) in Ningbo City, (2) two segments were selected within each of the selected townships, (3) 100 households were randomly selected from each segment, (4) one participant from each household was selected using a Kish grid. Finally, 6,336 valid questionnaires were collected.

### Tools used

We used the “Health Literacy Questionnaire of Chinese Citizens (2020),” which included three parts: personal characteristics, HL, and COVID-19 knowledge, published by the Chinese Center for Health Education. The first part aimed to collect personal characteristics such as gender, age, marital status, education, occupation, place of residence, annual household income, chronic conditions, and self-rated health (SRH).

The second part is the China Health Literacy Scale, which is used to evaluate HL (12). The 50-item scale includes three dimensions: knowledge and attitudes, behavior and lifestyle, and health-related skills. There are three types of questions on the scale: true or false (one point is awarded for correct response), single answer (one point is awarded for correct response), and multiple answers (two points are awarded for correct response). The overall Cronbach's α of the scale was 0.95 (13). Based on the data of this questionnaire survey, the Cronbach's α coefficient was calculated as 0.892. The maximum total score of the scale is 66 points. A total score of 53 (80% of 66) was considered adequate HL. A score of 0–52 was thought to indicate limited HL (14).

The third part is the COVID-19 knowledge Scale. The scale consisted of three dimensions, including (a) knowledge and, (b) and attitudes, and (c) behavior. For the three links of infectious diseases, the knowledge section has a total of 11 questions, covering the source of infection, route of transmission and susceptible people. It includes both single choice and multiple choice questions. The attitude part includes three aspects: the responsibility citizens should assume (6 questions, yes/no selection questions), the evaluation of COVID-19 related information reports (5 questions, five-level single-choice questions), and the evaluation of the government's prevention and control effectiveness (1 question, five-level single-choice questions). Seven questions in behavior section (yes/no choice questions), mainly including actively searching for relevant information or consulting medical staff. One point is awarded for a correct single-choice answer and two points are awarded for a correct multiple-choice answer. An overall score of 80% or more in the knowledge component indicates adequate COVID-19 knowledge.

The on-site survey was conducted by household survey, and the respondents were encouraged to complete the questionnaire by themselves. If the respondents could not complete the questionnaire independently, the investigators who had received unified training and passed the assessment completed the questionnaire by face-to-face inquiry. According to the above scheme, three stages of quality control methods are adopted: before, during and after the investigation. Before the survey, the Ningbo Center for Disease Control and Prevention completed the household sampling, coding and investigator training. In the survey, all counties and districts used the uniformly printed questionnaire. The investigator did not use inductive or suggestive language, reviewed the completion of the questionnaire on the spot, and finally filled in the name of the investigator and other survey completion information. After the investigation, the quality control personnel of each county and district shall review the questionnaires of each township in time.

### Statistical analysis

All data in this study were analyzed using SPSS 22.0 (SPSS Corp, Chicago, IL, USA). Two-tailed *P* value < 0.05 was considered statistically significant. COVID-19 knowledge and HL scores were dichotomized into two categories: adequate and limited. Pearson chi-square test was used to compare categorical variables between groups. The Mann-Whitney *U* test was used for comparison of ordinal data. Significant variables in chi-square test were included in multivariate logistic regression models. One model included HL and the other did not, the −2 log likelihood (−2LL) and Nagelkerke *R*^2^ changes were compared. The *R*^2^ mainly explains how much variation in COVID-19 knowledge can be attributed by the model. The −2LL can evaluate the model, and a smaller value indicates a better goodness of fit.

## Results

### COVID-19 knowledge level among different groups

As shown in Table 1, there were 6,336 subjects in this study, of which 24.8% had adequate HL. The male: female ratio was 1:1.05, and the average age was 49.55 ± 13.56 years. The Marital status was mainly married, accounting for 83.1% of the sample; The education level of the respondents was mainly Junior/Senior high school, accounting for 47.2% of the sample; With respect to occupation, the majority of participants (44.5%) were workers and farmers; 57.0% of the residents lived in rural areas; The number of people with household income (0–49,999) was the largest; The prevalence of chronic diseases was 25.9%; 70.1% of the residents thought that their health status was good.

**Table 1:** Association between COVID-19 knowledge and basic characteristics.

**Variables**		**N (%)**	**COVID-19 knowledge**		***χ^2^***	***P***
			**Limited**	**Adequate**		
	Total	6,336 (100.0)	5,344 (84.3)	992 (15.7)		
Gender	Male	3,092 (48.8)	2,638 (85.3)	454 (14.7)	4.334	**0.037**
	Female	3,244 (51.2)	2,706 (83.4)	538 (16.6)		
Age	15–34	1,074 (17.0)	808 (75.2)	266 (24.8)	115.671	**< 0.001**
	35–54	2,492 (39.3)	2,071 (83.1)	421 (16.9)		
	55–69	2,770 (43.7)	2,465 (89.0)	305 (11.0)		
Marital status	Single/widow/divorced	1,071 (16.9)	868 (81.0)	203 (19.0)	10.614	**0.001**
	Married	5,265 (83.1)	4,476 (85.0)	789 (15.0)		
Educational levels	Less than junior high school	1,867 (29.5)	1,710 (91.6)	157 (8.4)	254.551	**< 0.001**
	Junior/senior high school	2,990 (47.2)	2,571 (86.0)	419 (14.0)		
	College or above	1,479 (23.3)	1,063 (71.9)	416 (28.1)		
Occupation	Service/commercial	666 (10.5)	503 (75.5)	163 (24.5)	136.058	**< 0.001**
	Students	215 (3.4)	157 (73.0)	58 (27.0)		
	Workers/farmers	2,818 (44.5)	2,529 (89.7)	289 (10.3)		
	Others	2,637 (41.6)	2,155 (81.7)	482 (18.3)		
Place of residence	Rural areas	3,609 (57.0)	3,070 (85.1)	539 (14.9)	3.307	0.069
	Urban areas	2,727 (43.0)	2,274 (83.4)	453 (16.6)		
Annual household income	0–49,999	1,648 (26.1)	1,467 (89.0)	181 (11.0)	133.127	**< 0.001**
	50,000–99,999	1,595 (25.3)	1,413 (88.6)	182 (11.4)		
	100,000–149,999	1,344 (21.3)	1,129 (84.0)	215 (16.0)		
	≥150,000	1,719 (27.3)	1,312 (76.3)	407 (23.7)		
Chronic conditions	No	4,692 (74.1)	3,895 (83.0)	797 (17.0)	24.215	**< 0.001**
	Yes	1,644 (25.9)	1,449 (88.1)	195 (11.9)		
SRH	Good	4,441 (70.1)	3,724 (83.9)	717 (16.1)	2.990	0.224
	Fair	1,683 (26.6)	1,436 (85.3)	247 (14.7)		
	Poor	212 (3.3)	184 (86.8)	28 (13.2)		
Health literacy	Limited	4,762 (75.2)	4,291 (90.1)	471 (9.9)	482.576	**< 0.001**
	Adequate	1,574 (24.8)	1,053 (66.9)	521 (33.1)		

Boldface indicates statistical significance.

Table 1 demonstrates that 15.7% of the participants had adequate COVID-19 knowledge. The univariate analysis showed significant differences in COVID-19 knowledge by gender, age, marital status, educational, occupation, annual household income, chronic conditions, and HL. COVID-19 knowledge in females was slightly higher than that in males. COVID-19 knowledge gradually decreased with age. Interestingly, married people had lower COVID-19 knowledge than single people; People with higher education level and annual income had higher knowledge rate of COVID-19 than other groups. However, workers, farmers and patients with chronic diseases had limited COVID-19 knowledge. People with adequate HL had higher COVID-19 knowledge, and the difference reached 23.2%.

### Factors analysis using logistic regression model

Two logistic regression models were conducted to identify factors which might affect COVID-19 knowledge. Model 1 included significant variables (gender, age, marital status, educational level, occupation, annual household income, and chronic conditions) in univariate analysis, but did not include HL; and model 2 included HL. Changes in both models were evaluated by −2LL and R^2^. In model 1, gender, educational level, occupation, and annual household income were significantly associated with COVID-19 knowledge. The variation explained by the logistic regression model was Nagelkerke *R*^2^ = 0.074. In model 2, the Nagelkerke *R*^2^ was nearly doubled to 0.137 after HL was added to the model. And its −2LL is also reduced, indicating a better goodness of fit. People with adequate HL were the more likely to have adequate COVID-19 knowledge compared with those with limited HL (*OR* = 3.473, 95%CI = 2.974–4.057, *P* < 0.001). HL is the most important factor likely to affect COVID-19 knowledge, compared to other factors.

### Differences in knowledge of COVID-19 prevention and control among limited and adequate HL residents

As shown in Table 3, except for the choice of mask, there was no significant difference between the two groups, the awareness rate of COVID-19 knowledge in the adequate HL group was higher than that in the limited HL group (*P* < 0.001). The least correct question was the route of transmission of the COVID-19, and the most correct was the shortest quarantine period.

### Differences in attitude of COVID-19 prevention and control among limited and adequate HL residents

Table 4 summarizes the survey results of the three aspects of prevention and control attitudes. In terms of each prevention and control attitude, the selection results of each question in the adequate HL group were more positive than those in the limited HL group (*P* < 0.001). Those who with adequate HL were more likely to recognize the responsibility of citizens for epidemic prevention and control of infectious diseases, more likely to agree the release and report of COVID-19 related information, and more likely to recognize the achievements made by the government in epidemic prevention and control of COVID-19.

### Differences in behavior of COVID-19 prevention and control among limited and adequate HL residents

As shown in Table 5, the limited HL group was more likely to use the telephone, while the adequate HL group was more likely to use the internet for information query (*P* < 0.05). There was no significant difference between the two groups in using the Internet to consult doctors.

### Analysis of correlation

We analyzed the relationship between the score of HL, the score of COVID-19 knowledge and other dimension, and found that the score of HL was positively correlated with each part, among which the correlation coefficient of knowledge was the largest, and behavior was the smallest (*P* < 0.001).

## Discussion

### Principal findings

The COVID-19 pandemic has stirred fear because its direct impact on the public has created unprecedented challenges for education and healthcare systems (15). As vaccination remains slow and specific treatments are lacking, non-pharmaceutical public health interventions have become important in the fight against COVID-19 (16).

HL has been shown to be associated with a variety of diseases (17, 18), but its relationship with COVID-19 knowledge is still poorly studied. We explored the relationship between them through a large sample cross-sectional study. As shown in Tables 1, 2, our study showed that people with adequate HL had higher COVID-19 knowledge, and the difference reached 23.2%. And after adjusting for a series of confounding factors, the *OR* value of HL still reached 3.473, and the Nagelkerke *R*^2^ increased nearly doubled. HL is an important influencing factor of COVID-19 knowledge. In addition, gender, age, short marital status, educational level, occupation, annual household income, and chronic conditions also affected COVID-19 knowledge in our study. The elderly people, people with low education level, workers and farmers, and people with low income have more limited COVID-19 knowledge. This is consistent with the conclusion of the study by Jaber et al. (19). Another study found that COVID-19 was connected with lower HL in rural areas (20). The government should take different intervention measures for different groups. But there is no doubt that HL is the easiest and most rapid modifiable factor.

**Table 2:** Logistic regression model of factors influencing adequate COVID-19 knowledge.

**Variables**	**Model 1**			**Model 2**		
	***OR***	**95% CI**	***P***	***OR***	**95% CI**	***P***
Gender (ref. = Male)	1.163	1.01–1.339	**0.036**	1.170	1.012–1.352	**0.034**
**Age (ref**. = **15–34)**						
35–54	0.968	0.783–1.196	0.764	1.033	0.83–1.287	0.769
55–69	0.913	0.707–1.18	0.488	1.083	0.83–1.412	0.558
Marital status (ref. = single/widow/divorced)	0.967	0.783–1.195	0.755	0.913	0.735–1.134	0.410
**Educational levels (ref**. = **Less than junior high school)**						
Junior/senior high school	1.517	**1.222–1.884**	**< 0.001**	1.353	1.085–1.687	**0.007**
College or above	2.812	**2.136–3.702**	**< 0.001**	1.924	1.444–2.564	**< 0.001**
**Occupation (ref**. = **Service/Commercial)**						
Students	1.368	0.902–2.074	0.140	1.332	0.866–2.048	0.192
Workers/Farmers	0.765	0.594–0.986	**0.038**	0.880	0.677–1.144	0.341
Others	0.946	0.764–1.171	0.610	1.046	0.839–1.305	0.689
**Annual household income (ref**. = **0–49,999)**						
50,000–99,999	0.909	0.726–1.139	0.408	0.914	0.727–1.15	0.444
100,000–149,999	1.097	0.873–1.378	0.427	1.078	0.853–1.361	0.529
≥150,000	1.376	1.102–1.719	**0.005**	1.320	1.051–1.657	0.017
Chronic conditions (ref. = No)	1.012	0.837–1.223	0.903	1.030	0.85–1.25	0.761
Health literacy (ref. = limited)	–	–	–	3.473	2.974–4.057	**< 0.001**
−2LL	5,186.791			4,942.475		
Nagelkerke *R*^2^	0.074			0.137		

Boldface indicates statistical significance.

In addition, HL is closely related to the three dimensions of COVID-19. Compared with the limited HL group, the adequate HL group had a higher rate of COVID-19 knowledge (Table 3), a more positive attitude (Table 4), a more active behavior (Table 5). Interestingly, in terms of behavior, those in limited HL group were more likely to use the phone to get information, while those in adequate HL group were more likely to use the internet. Public intervention measures based on HL can help to promote COVID-19-related health behaviors and reduce the risk of COVID-19 infection among college students (21). The results of correlation analysis showed that HL had the strongest association with COVID-19 knowledge and the weakest association with COVID-19 behavior (Table 6). We must not only understand the knowledge, but also translate the knowledge gained into health-promoting behaviors to improve or maintain health (22, 23). With sufficient knowledge of COVID-19, change your emphasis on COVID-19 and act accordingly to protect yourself.

**Table 3:** Comparison of knowledge among people with and without HL.

**Question**	**Health literacy**		***χ^2^***	***P***
	**Limited**	**Adequate**		
K1 correct *n* (%)	1,839 (38.3)	885 (56.2)	155.651	< 0.001
K2 correct *n* (%)	4,117 (85.7)	1,515 (96.2)	126.574	< 0.001
K3 correct *n* (%)	2,076 (43.2)	955 (60.6)	144.506	< 0.001
K4 correct *n* (%)	1,382 (28.8)	684 (43.4)	116.536	< 0.001
K5 correct *n* (%)	1,146 (23.9)	506 (32.1)	42.348	< 0.001
K6 correct *n* (%)	1,835 (38.2)	994 (63.1)	298.516	< 0.001
K7 correct *n* (%)	2,992 (62.3)	1,397 (88.7)	385.967	< 0.001
K8 correct *n* (%)	3,012 (62.7)	965 (61.3)	1.011	0.315
K9 correct *n* (%)	1,751 (36.4)	999 (63.4)	352.270	< 0.001
K10 correct *n* (%)	4,108 (83.6)	1,533 (97.3)	197.277	< 0.001
K11 correct *n* (%)	3,242 (67.5)	1,470 (93.3)	410.881	< 0.001

K1, Body temperature over how much need to go to the hospital; K2, What is the minimum number of days of isolation after close contact; K3, People susceptible to the COVID-19; K4, The source of infection of the COVID-19; K5, Route of transmission of the COVID-19; K6, Personal protective measures; K7, The proper way to wash hands; K8, Choice of mask; K9, Proper way to wear masks; K10, The correct way to eat in a group; K11, Protective measures in low-risk areas.

**Table 4:** Comparison of attitude among people with and without HL.

**Attitude question**		**HL limited**					**HL adequate**					**Statistical tests**	
		**No** ***n*** **(%)**		**Yes** ***n*** **(%)**			**No** ***n*** **(%)**		**Yes** ***n*** **(%)**			*χ^2^*	***P***
Responsibility	AA1	547 (11.4)		4,258 (88.6)			18 (11.1)		1,557 (98.9)			154.131	< 0.001
	AA2	614 (12.8)		4,191 (87.2)			19 (1.2)		1,556 (98.8)			177.733	< 0.001
	AA3	572 (11.9)		4,233 (88.1)			16 (1.0)		1,559 (99.0)			168.080	< 0.001
	AA4	788 (16.4)		4,017 (83.6)			27 (1.7)		1,548 (98.3)			229.582	< 0.001
	AA5	1,008 (21.0)		3,797 (79.0)			41 (2.6)		1,534 (97.4)			291.517	< 0.001
	AA6	1,262 (26.3)		3,543 (73.7)			44 (2.8)		1,531 (97.2)			401.376	< 0.001
**Evaluation of information**		**Complete agreement**	**Agree**	**General**	**Disagree**	**Complete disagreement**	**Complete agreement**	**Agree**	**General**	**Disagree**	**Complete disagreement**	***Z***	***P***
	AB1	59.5%	36.2%	2.6%	0.4%	0.4%	73.5%	25.6%	0.8%	0.0%	0.1%	9.997	< 0.001
	AB2	53.9%	39.8%	4.1%	0.6%	0.6%	70.4%	28.3%	1.1%	0.1%	0.1%	11.759	< 0.001
	AB3	49.2%	42.2%	6.3%	0.8%	0.6%	65.3%	32.1%	2.2%	0.1%	0.2%	11.565	< 0.001
	AB4	47.5%	42.4%	7.5%	0.9%	0.7%	65.4%	31.9%	2.3%	0.3%	0.1%	12.932	< 0.001
	AB5	45.9%	42.7%	8.3%	1.2%	1.0%	65.1%	31.8%	2.7%	0.1%	0.2%	13.957	< 0.001
**Evaluation of policies**		**Very good**	**Good**	**General**	**Poor**	**Very poor**	**Very good**	**Good**	**General**	**Poor**	**Very poor**	***Z***	***P***
	AC	69.3%	28.6%	1.8%	0.3%	0.0%	79.4%	19.8%	0.5%	0.3%	0.0%	7.843	< 0.001

AA1, Timely report of patients found; AA2, Cooperate with the government's investigation work and report the situation truthfully; AA3, Cooperate with health institutions to implement quarantine observation, treatment and other measures; AA4, Do not touch, buy or eat wild animals; AA5, No rumor, no price gouging; AA6, No discrimination against infectious disease patients, suspected patients and pathogen carriers. AB1, Timely, open and transparent publication of information; AB2, Scientific and authoritative sources of information; AB3, Sufficient information reporting; AB4, The presentation of information is easy to understand; AB5, Informative guidance. AC, Evaluation of the effectiveness of government prevention and control.

**Table 5:** Comparison of behavior among people with and without HL.

**Question**	**Health literacy**		***χ^2^***	***P***
	**Limited**	**Adequate**		
B1 practice *n* (%)	568 (11.8)	137 (8.7)	11.767	0.001
B2 practice *n* (%)	387 (8.1)	96 (6.1)	6.505	0.011
B3 practice *n* (%)	1,833 (38.1)	503 (31.9)	19.718	< 0.001
B4 practice *n* (%)	2,397 (49.9)	1,286 (81.7)	490.475	< 0.001
B5 practice *n* (%)	1,918 (39.9)	1,133 (71.9)	487.388	< 0.001
B6 practice *n* (%)	555 (11.6)	302 (19.2)	59.295	< 0.001
B7 practice *n* (%)	468 (9.7)	178 (11.3)	3.179	0.075

B1, Call 12320 for information; B2, Call the psychological counseling hotline; B3, Telephone or on-site consultation with community doctors; B4, Use the internet to search disease prevention knowledge; B5, Use the internet to check the epidemic situation near your residence; B6, Use the internet to query the information of fellow passengers; B7, Use the internet to consult a doctor.

**Table 6:** Correlation analysis between HL score and COVID-19 score.

**Variable**	**COVID-19 knowledge score**	**COVID-19 attitude score**	**COVID-19 behavior score**
HL score	0.631^*^	0.503^*^	0.230^*^

^*^P < 0.001.

HL is a broad and important topic in public health, yet it is still underestimated globally and thus considered a silent epidemic (24). The COVID-19 pandemic has been accompanied by rapidly emerging evidence, changing guidelines, and misinformation, posing new challenges to HL (10). There are widespread misconceptions about COVID-19 transmission and protection. Most people are unaware that asymptomatic infected persons can transmit the virus. In this survey, the correct rate of questions about the source of infection was only 32.6%. People with limited HL had a poorer understanding of COVID-19 symptoms, were less able to identify behaviors to prevent infection, were more likely to endorse misinformed beliefs about COVID-19 and vaccinations, and experienced more difficult finding and understanding the government's message on COVID-19 (25). Another study also showed that acceptance of COVID-19 vaccines is associated with the ability to detect fake news and HL (26).

Therefore, in these uncertain and difficult times, good HL has never been more vital for survival. Factors such as age, sex, chronic disease, place of residence and economic status can affect health literacy (27, 28). Adherence to protective measures is an important component of controlling the COVID-19 pandemic, and HL is a major driver of this adherence (29). The results of this study not only provide new evidence for understanding the importance of residents' HL, but also point out the key points for carrying out health education targeted to cope with the threat of sudden major infectious diseases and make up for the shortcomings of residents' HL. Targeted interventions and strategies should be developed to strengthen the HL of the population and improve people's attitudes, so as to reduce the spread of COVID-19.

## Limitations

This study has some limitations. The results of this study are only from the survey in Ningbo City, Zhejiang Province, and cannot represent the situation of the whole Zhejiang province, let alone the whole of China. At the same time, we were unable to compare knowledge rates of COVID-19 among countries, as the type and difficulty of questionnaires used varied across countries. Due to the cross-sectional design of this study, it cannot prove a causal relationship between HL and COVID-19 knowledge. Future studies should examine these relationships closely. And the process of how HL affects COVID-19 knowledge needs to be further studied. In addition, logistic regression analysis only controlled for sociodemographic factors, and there were other factors that would affect the relationship between HL and COVID-19 knowledge.

## Conclusions

This study showed that knowledge of COVID-19 was associated with HL. Furthermore, gender, age, marital status, educational, occupation, annual household income, and chronic conditions are associated with COVID-19 knowledge. Targeted health education and promotion strategies should be adopted for different populations to improve the HL of residents, so as to reduce the spread of COVID-19.

## Data availability statement

The raw data supporting the conclusions of this article will be made available by the authors, without undue reservation.

## Ethics statement

This study was reviewed and approved by the Research Ethics Committee of the Ningbo Municipal Center for Disease Control and Prevention (Approval No.: 202203). Informed consent was obtained from all subjects involved in the study.

## Author contributions

ZT and QX were responsible for conceptualization, data collection, methodology, resources, and software. YZ and SD were responsible for conceptualization, data collection, and methodology. QM was responsible for methodology and software. HF and QJ were responsible for data collection and investigation. YD was responsible for conceptualization, funding acquisition, resources, supervision, reviewing, and editing the manuscript. All authors contributed to the critical revision of the manuscript for important intellectual content, review, and approval of the final manuscript.
